# Host coevolution alters the adaptive landscape of a virus

**DOI:** 10.1098/rspb.2016.1528

**Published:** 2016-09-28

**Authors:** Alita R. Burmeister, Richard E. Lenski, Justin R. Meyer

**Affiliations:** 1 Department of Microbiology and Molecular Genetics, Michigan State University, East Lansing, MI, USA; 2 Program in Ecology, Evolutionary Biology and Behavior, Michigan State University, East Lansing, MI, USA; 3 BEACON Center for the Study of Evolution in Action, Michigan State University, East Lansing, MI, USA; 4 Division of Biological Sciences, University of California, San Diego, La Jolla, CA, USA

**Keywords:** adaptive landscape, coevolution, experimental evolution, key innovation, viruses

## Abstract

The origin of new and complex structures and functions is fundamental for shaping the diversity of life. Such key innovations are rare because they require multiple interacting changes. We sought to understand how the adaptive landscape led to an innovation whereby bacteriophage λ evolved the new ability to exploit a receptor, OmpF, on *Escherichia coli* cells. Previous work showed that this ability evolved repeatedly, despite requiring four mutations in one virus gene. Here, we examine how this innovation evolved by studying six intermediate genotypes of λ isolated during independent transitions to exploit OmpF and comparing them to their ancestor. All six intermediates showed large increases in their adsorption rates on the ancestral host. Improvements in adsorption were offset, in large part, by the evolution of host resistance, which occurred by reduced expression of LamB, the usual receptor for λ. As a consequence of host coevolution, the adaptive landscape of the virus changed such that selection favouring four of the six virus intermediates became stronger after the host evolved resistance, thereby accelerating virus populations along the path to using the new OmpF receptor. This dependency of viral fitness on host genotype thus shows an important role for coevolution in the origin of the new viral function.

## 1. Introduction

The diversity of complex structures and functions in the living world is striking. However, a detailed understanding of how species evolve key innovations is difficult because of limitations of both theory and data [1]. One challenge is to understand how populations evolve traits that require multiple interacting changes, such as specific deformations in the reactive pocket of an enzyme [2] or the specialized modifications of an appendage [3]. If each intermediate genotype leading to the new function is more fit than its immediate predecessor, then the new function can evolve readily, even if the fitness benefits do not accrue from the new function *per se* [4]. By contrast, the evolution of a new function will be slower and more difficult, although not impossible [5], if any of the requisite intermediates have lower fitness than their progenitors.

Evolution experiments provide a powerful way to study how mutations and the environment together can lead to key innovations. For example, the evolution of the new ability of *Escherichia coli* to consume citrate in an experimental population depended on its history, including the appearance of earlier potentiating mutations, as well as on the selective environment [6,7]. Experimental manipulation of environmental conditions can also provide information on factors involved in the evolution of novel functions. For example, Bono *et al*. [8] showed that increasing competition led a virus to evolve the ability to infect a new host. In another study on viruses, Herfst *et al.* [9] found that the genetic background of influenza influenced whether viral populations evolved the ability to spread via airborne transmission. Although genetic and environmental manipulations can reveal factors important for the evolution of new traits, the selective pressures that favour intermediate genotypes often remain elusive. One limitation to the study of innovations, even in the laboratory, is their rare occurrence; therefore, having parallel histories leading to the same innovation would increase one's ability to analyse how and why new traits evolve. In the ideal scenario, the selective benefit or cost for each intermediate mutation along multiple independent paths leading to the same innovation could be described quantitatively, in terms of the resulting fitness, and qualitatively in terms of the phenotypic traits responsible. In this study, we use this framework to describe how replicate populations of the virus bacteriophage λ evolved a key innovation in parallel, and we specifically investigate how the host's coevolution changed the adaptive landscape for the virus, sometimes potentiating the key innovation.

Meyer *et al.* [10] previously reported the parallel evolution of a new function while studying populations of bacteriophage λ in the laboratory. They found that λ sometimes evolved to target a new receptor on the surface of its host, the bacterium *E. coli*, with the innovation occurring in 24 of 96 replicate populations. They further showed that these parallel innovations involved a common set of four mutations in the phage's host-specificity gene. The ancestral phage λ adsorbs to and infects through a porin protein called LamB; the evolved phage that acquired the key innovation can also use another porin called OmpF. This new function had not previously been observed, despite decades of intensive study of λ by virologists and molecular biologists. One reason this new function had not previously been observed was that it required four mutations in the *J* gene [10]. *J* encodes the tail protein, J, which λ uses to recognize and attach to LamB receptor proteins on the surface of the host cell. Importantly, none of the phage genotypes examined by Meyer *et al.* [10] that had just three of the four required mutations had any capacity whatsoever to infect hosts that expressed only the OmpF receptor. It would be extremely unlikely to detect a function that required four simultaneous mutations using traditional microbiological methods. However, Meyer *et al.* [10] observed the mutations accumulate over time as the phage and bacteria coevolved. In fact, the new function emerged repeatedly and quickly, arising in 24 of 96 populations after only 12 days on average. Here, we address the question as to how the phages evolved this new function, and so quickly, if the intermediate genotypes did not confer any ability to exploit the alternative receptor.

One compelling hint is the highly non-random pattern of substitutions observed among the independently derived *J* alleles. In particular, all of the phage that could infect through the OmpF receptor had mutations in four narrow regions of the *J* gene [10]. We know that the four mutations did not arise simultaneously because alleles with subsets of these mutations were sampled at earlier time points [10]. The genetic parallelism evident in the intermediate states strongly suggested that natural selection favoured the intermediate *J* alleles, even though they did not allow the phage to use the OmpF receptor. Meyer *et al.* [10] also observed that the phage mutations accumulated while the hosts were evolving substantial, but not complete, resistance through reduced expression of the LamB receptor. This synchrony suggested that the reduction in the density of the LamB receptor increased the strength of selection on λ to improve its binding to LamB, thereby favouring certain mutations in *J* that, fortuitously, served as stepping stones on the way to the new capacity to use the OmpF receptor. Under this scenario, the arms race between the coevolving hosts and parasites changed the fitness landscape for the phage populations, thereby opening new, and uphill, paths to the innovation [11]. Taken together, these observations and hypotheses lead to the predictions that we test here.

First, we test whether antagonistic coevolution caused alternate steps that increased host fitness at the expense of parasite fitness and vice versa [12]. For the coevolved λ and *E. coli*, we expect adsorption rates to change with the phages' and hosts' coevolutionary steps. More specifically, we predict that: (i) the ancestor phage should adsorb faster to the ancestral cells than to the coevolved cells, (ii) the evolved phages should adsorb faster to the ancestral cells than to the coevolved cells, (iii) the evolved phages should adsorb faster than the ancestor phage to the ancestral cells, and (iv) the evolved phages should adsorb faster than the ancestor phage to the coevolved cells.

Second, we test the hypothesis that the coevolutionary arms race changed the phage's adaptive landscape, opening new and uphill paths to the key innovation. That is, we predict that the evolved intermediate phage genotypes should have a fitness advantage over the ancestral phage. More specifically, these intermediates should possess a greater advantage when competing for coevolved host cells than when competing for ancestral hosts; in the most extreme case, the intermediate phage types might have an advantage only on the coevolved host cells.

To test these two sets of predictions, we measured the adsorption rates of six independently evolved phage λ intermediates. We also measured the fitness of each intermediate in competition with the ancestral phage. To examine the effects of host coevolution, we conducted all of the assays using both the ancestral sensitive *E. coli* cells and resistant coevolved cells. The resulting data generate the phage genotype-by-host (GxH) reaction norm for phage fitness.

## 2. Material and methods

### (a) Overview

In the coevolution experiments performed by Meyer *et al.* [10], strictly lytic phage λ strain cI26 and *E. coli* B strain REL606 (electronic supplementary material, table S1) were propagated together in 96 replicate communities by transferring 0.1 ml from each 10 ml culture into a new flask containing 9.9 ml of fresh medium every day for 20 days. Whenever possible, we performed our assays under the same conditions as those used in that earlier study; however, as discussed below, we sometimes had to alter certain conditions in order to obtain reliable measurements. Before each transfer, a separate 0.2 ml mixed-population sample (i.e. with phage and host together) was frozen; these samples provide viable representatives of the λ and *E. coli* populations that were used to understand their evolution. Meyer *et al.* found that the first mutations to reach high frequency in the bacterial populations were in *malT*, which encodes a positive regulator of *lamB*, which in turn encodes the native phage receptor protein LamB. These mutations reduced expression of LamB, thereby conferring substantial (but not complete) host resistance to the ancestral phage, and they reached near-fixation in all host populations by day 8 of the experiment. Also, one-quarter (24 out of 96) of the phage populations evolved the new ability to infect the bacteria through the OmpF protein, which took 12 days on average (range: 9–18 days).

To study the adsorption rate and competitive fitness of the intermediate λ genotypes, we sampled a single phage isolate from six independently evolved populations exactly four days before the ability to use OmpF was first observed in that population; we confirmed that none of these phages had the ability to infect through OmpF. We chose the six populations haphazardly, except that we paid attention to choosing phage from replicates that had evolved distinct *J* alleles by the end of the original experiment. We sequenced the *J* genes of the six intermediate genotypes to check that they had some, but not all, of the four mutations needed to exploit OmpF.

We measured phage adsorption rates because the J protein physically interacts with host receptors to initiate infections and mutations in the *J* gene affect adsorption to the host's LamB receptors [13,14]. Thus, changes in the adsorption rate would help elucidate the mechanism of phage adaptation. We also directly assayed relative fitness in a manner similar to that used in studies of *E. coli* evolution [15], where evolved genotypes compete head-to-head against a genetically marked ancestor. We performed all adsorption rate and fitness assays in two environments that differed only in the identity of the bacterial host. One environment used the sensitive ancestral host, REL606; the other used a partially resistant evolved strain, EcC4, that has a point mutation in *malT*, which generates a premature stop codon in the gene at amino acid position 295.

### (b) Culture conditions

All *E. coli* cultures were inoculated from freezer stocks into Luria-Bertani (LB) broth [16], then grown overnight at 37°C while shaking at 120 r.p.m. Host cells were preconditioned for the phage competition experiments by growing them in the same medium and other conditions used in the coevolution experiments performed by Meyer *et al.* [10]: 10 ml of modified M9 (M9 salts with 1 g l^−1^ magnesium sulfate, 1 g l^−1^ glucose and 0.02% LB) for 24 h at 37°C and 120 r.p.m. Phage strains were isolated by plating diluted frozen samples on a lawn of REL606 cells suspended in soft agar (LB with 0.8% w/v agar) and spread on the surface of an LB plate (LB broth with 1.6% w/v agar). We then picked an individual plaque (a small clearing in the host lawn caused by the phage population expansion from a single infection) for each phage strain of interest. These isolates were immediately re-suspended in soft agar and the plaque-picking procedure was repeated to ensure that only a single phage genotype was sampled. Next, the phage stocks were grown on approximately 10^8^ cells of REL606 for approximately 16 h in the same modified M9 medium and other conditions as above. The phage were then harvested by chloroform preparation [17] and stored at 4°C. Samples of each isolated phage were also preserved at −80°C in 15% v/v glycerol.

### (c) Sequencing the *J* gene

We sequenced the *J* gene (host-specificity gene, GenBank: NP_040600) from all six λ intermediates; previous work showed that the mutations responsible for the ability to use OmpF were in this gene [10]. The sequences were obtained by the Michigan State University Research Technology Support Facility using an ABI 3730xl Sanger sequencing platform. PCR-amplified fragments of the *J* gene were sequenced after column-purification with the GE Illustra GFX kit; electronic supplementary material, table S1, shows the primers used for amplification and sequencing. Point mutations were automatically scored using the DNASTAR SeqMan SNP analysis tool and then manually confirmed.

### (d) Sequencing the *cI* gene

Phage λ cI26, the ancestral virus in this study, has a 1 bp deletion in *cI* (repressor gene, GenBank: NP_0040628.1), causing a frame shift that makes it obligately lytic. We sequenced the *cI* gene using the same general methods as for the *J* gene and the specific primers shown in electronic supplementary material, table S1.

### (e) Adsorption assays

We used a modified version of the 96-well filter plate method described by Shao & Wang [18] while keeping other conditions as close as feasible to the Meyer *et al.* [10] evolution experiment. In preliminary experiments, we found that measuring adsorption rates in these conditions required long periods, such that phage replication confounded the measurements. Therefore, we added chloramphenicol to inhibit cell growth and thereby prevent phage replication. We diluted each phage stock to 2 × 10^5^ plaque-forming units per millilitre in modified M9, added 10 µl of a solution containing 15 µg ml^−1^ chloramphenicol in ethanol, and then put 550 µl of each resulting phage mixture into one well of a ClavePak Racked Tubes block (Denville Scientific no. B1251-S) for every replicate assay. The ClavePak block was then incubated for 20 min at 37°C and 120 r.p.m. Immediately before adding host cells (designated as *T*_0_), we transferred 100 µl to a 96-well filter plate (Pall no. 8019) that was centrifuged for 2 min at 2000 r.p.m. In total, 50 µl of each host type or blank cell-free medium was then added to each well, the contents were gently mixed by pipetting, and the plate was incubated at 37°C and 120 r.p.m. The resulting phage concentration during the assay was 1.8 × 10^5^ plaque-forming units per millilitre, and the host-cell density was 8.6 × 10^7^ colony-forming units per ml for each host type. After 67 min, we sampled and processed 100 µl from each well using the same procedures as at *T*_0_. The background decay rate of each phage genotype was estimated as the rate at which free phage declined when the blank cell-free medium was added instead of host cells. The phage adsorption rate was estimated as the rate at which phage disappeared after subtracting the background decay rate, and then dividing by the density of cells during the adsorption-rate assay. The mean background decay rate was 0.29 h^−1^ (electronic supplementary material, figure S1). This value is greater than one previously reported for phage λ [19], which may reflect different assay conditions, the specific phage genotypes tested, or both.

### (f) Phage fitness assays

We performed phage fitness assays by direct competitions between each of the six evolved λ isolates and a genetically marked ancestor phage that can be differentiated when grown on a lawn of *E. coli* cells. Some aspects of the assays had to be refined before reliable estimates could be obtained, as discussed in the electronic supplementary material. Here, we provide the methods used in the final approach and some background on protocol development.

First, we modified the ancestral λ strain to make blue plaques on a lawn of *lacZα*^−^ *E. coli* (strain DH5α) supplemented with X-gal (5-bromo-4-chloro-3-indolyl-β-d-galactopyranoside). When *E. coli* cells metabolize X-gal, they produce a blue compound; however, cells without a functional *lacZα* gene cannot metabolize X-gal. By inserting a functional *lacZα* gene into the phage genome, the phage complements the cell's capacity to metabolize X-gal and generates a blue spot where a plaque forms. To construct a marked version of phage λ strain cI26, we infected *E. coli* cells that contained a plasmid encoding the phage *R* gene fused to *lacZα* [18,20]. Phage λ has an efficient system for homologous recombination [21], allowing some of the resulting genomes to recombine with the *R*-*lacZα* fusion. The phage progeny were then plated on a lawn of DH5α hosts in the presence of X-gal, and a single blue plaque was chosen. Upon studying this marked strain, we found that the marker was not neutral but instead reduced phage fitness. To quantify the marker's fitness cost, we computed the mean ±95% confidence intervals of the selection rate against the marker across several blocks of experiments on each host (ancestral host REL606: 0.120 h^−1^ ± 0.019 based on six blocks; evolved host EcC4: 0.039 h^−1^ ± 0.017 based on four blocks). The marker effect varied by host (*F*_1,54_ = 46.4, *p* < 0.001) but not by block (*F*_5,54_ = 1.395, *p* = 0.241), which we account for as described below in the sections on Statistical analyses and Results. A fitness cost was also reported for this marker in a related λ strain when phage competed for *E. coli* K12 hosts [18].

The phage fitness assays were run in the same medium and other conditions as used in the coevolution experiments performed by Meyer *et al.* [10]. For each of the six phage isolates tested, we grew one stock culture under the conditions described above. We used aliquots from that stock culture to run replicate assays on both the ancestral (REL606) and evolved (EcC4) hosts. Competitions were carried out in sets of eight assays per host, except for phage B2, which was run with four assays per host; procedural errors led to occasional missing values. The assays were blocked such that all replicates for one phage genotype were assayed on both hosts on the same day. To initiate a competition, we added approximately 10^8^ *E. coli* cells (REL606 or EcC4) acclimated to the culture conditions, approximately 10^5^ of the marked ancestral phage λ_lacZ_, and approximately 10^5^ of a particular evolved λ genotype to each flask. The host density was chosen to match the start of the coevolution experiments performed by Meyer *et al.* [10]; however, phage densities were almost 10-fold lower because we could not produce dense stocks of the evolved types, despite several attempts. (The lower density should reduce competition between the phage genotypes and thereby diminish our ability to detect fitness differences. Nonetheless, we measured significant fitness gains for the evolved phage, as shown in the Results.) The mixed cultures were incubated for 8 h, and the phage were sampled and enumerated at the beginning and end of that period. We chose a duration of 8 h for two reasons: (i) given the large fitness advantages of the evolved genotypes, they would drive the ancestral phage to very low relative frequency if the assays ran for 24 h (the period between transfers during the original evolution experiment), thereby compromising our ability to quantify the fitness difference, and (ii) resistant host mutants often arose and reached substantial frequency in 24 h, thereby altering the competitive environment of the phage. We discuss these time constraints more fully in the electronic supplementary material. Phage samples were diluted in TM (50 mM tris hydrochloride, pH 7.5 and 8 mM magnesium sulfate), and multiple dilutions were plated to find one where individual plaques could be counted. Plates used to count the phage contained DH5α host cells and 9.5 mg ml^−1^ X-gal in the top agar, and they were incubated at 37°C for 2 days to allow the blue plaques to develop full pigmentation.

We calculated fitness for the phage using the difference, not the ratio, of the two competitors' realized growth rates during the competition assay. This quantity, sometimes called the selection rate *r*, is related to, but distinct from, the more familiar ratio of the competitors' growth rates (i.e. relative fitness *W*). We used the difference here because it is more robust to large differences in growth rates between competitors, including those cases where one competitor declines and the other increases in abundance [22,23], which we sometimes observed.

### (g) Statistical analyses

We tested the following four predictions based on the adsorption-rate assays: (i) the ancestor phage adsorbs faster to the ancestral cells, REL606, than to the coevolved cells, EcC4, (ii) the evolved phages adsorb faster to the ancestral cells than to the coevolved cells, (iii) the evolved phages adsorb faster than the ancestor phage to the ancestral cells, and (iv) the evolved phages adsorb faster than the ancestor phage to the coevolved cells. Given the predicted directional effects, we used one-tailed *t*-tests of the associated hypotheses.

For the competitive fitness assays, we expected the fitness of the evolved phage (expressed as a selection rate differential relative to the marked ancestral phage) to be greater on the coevolved host cells than on the ancestral cells. We also expected the realized growth rates of the evolved phage to be lower on the coevolved cells than on the ancestral cells. Therefore, we employed one-tailed *t-*tests of these hypotheses. (In those cases where the outcome was opposite to our expectation, we report the *p*-value as >0.5.) We tested each hypothesis for six evolved phage genotypes, and so we performed Bonferroni corrections for multiple comparisons, resulting in an adjusted *α* = 0.05/6 = 0.0083 per test.

We performed a two-way ANOVA to determine the effect of assay date (block) and host type (coevolved versus ancestral) on the fitness cost of the phage's *lacZα* marker. This analysis showed that the *lacZα* marker imposed a greater cost when competing for the ancestral cells than for the coevolved cells. Therefore, we also asked whether correcting for this effect would alter our interpretation of the differences in phage fitness on the two host types; the correction was a simple subtraction of the grand mean of the difference in the fitness cost of the marker between the two hosts. In fact, this correction did not affect our interpretation (electronic supplementary material, table S2).

All analyses were performed in R v. 3.1.1 [24] using custom scripts.

## 3. Results and discussion

### (a) Mutations in the *J* and *cI* genes of the evolved phage intermediates

The six independently derived λ genotypes that we studied had different subsets of the four *J* mutations needed to exploit the OmpF receptor (table 1), indicating that they were intermediates; that is, each phage had some, but not all, of the mutations associated with that new function. In addition, all six phages had one or more other mutations in the *J* gene that were not required to use OmpF (table 1). In four cases (A7, B2, D9 and G9), the additional mutations were also present in the eventual OmpF^+^ phage (i.e. the phage with the new ability to use the OmpF receptor), indicating that these genotypes were on the evolutionary line of descent leading to that innovation. In two cases (A12 and E4), the other mutations were not present in the later OmpF^+^ isolates, implying that these genotypes were somewhat off the direct line of descent leading to the OmpF^+^ phage. We also sequenced the *cI* repressor gene to ensure that the evolved phages did not contain *cI* reversions, which would allow them to undergo lysogeny and replicate while integrated into the host chromosome. Five of the evolved phages had no new mutations in that gene. Phage A12 had a point mutation (G170A) in the out-of-frame portion of the gene, where it should not affect the potential for lysogeny.

**Table 1.** Mutations in the *J* gene of the evolved phage λ isolates in this study. (Each virus was sampled from a different source population 4 days before phage that could use the OmpF receptor were first detected. Each mutation is identified by its ancestral DNA base, its position in the *J* gene, and the evolved base. Asterisks under ‘line of descent’ indicate a mutation was present in the later phage able to use OmpF. Meyer *et al*. [10] found that phage λ requires four mutations—one or more in four specific regions of *J* to use OmpF: (A) one or more mutations between positions 2969 and 2999, (B) a mutation at 3320 or 3321, (C) the specific mutation G3319A and (D) the specific mutation A3034G. The last column shows which, if any, of these categories each mutation satisfies.)

source population	day of isolation	set of *J* mutations	line of descent	OmpF category
A12	10	C599T	*	
		G2921A		
		T2991G	*	A
		C3033T		
		C3147A		
		T3380C	*	
A7	10	A2989G	*	A
		C2999T	*	A
		C3119T	*	
		G3319A	*	C
B2	13	C2969T	*	A
		C3119T	*	
		G3319A	*	C
D9	8	C2879T	*	
		T2991G	*	A
		C3119T	*	
		G3319A	*	C
E4	13	C2969T		A
		C3119T	*	
		G3319A	*	C
G9	11	A1747G	*	
		A2989G	*	A
		C2999T	*	A
		C3119T	*	
		G3319A	*	C

### (b) Phage evolution increases and host coevolution reduces adsorption rates

Phage λ uses its J protein to adsorb to the LamB receptors on the surface of host cells [25], and the mutations that distinguish the intermediate phage genotypes from their ancestor are in the gene that encodes that protein. Therefore, we hypothesize that adaptive evolution of the phage populations led to increased rates of adsorption to LamB prior to the emergence of the ability to exploit the alternative OmpF receptor, which would explain why the intermediates evolved even before they had acquired that new ability.

**Figure 1.**
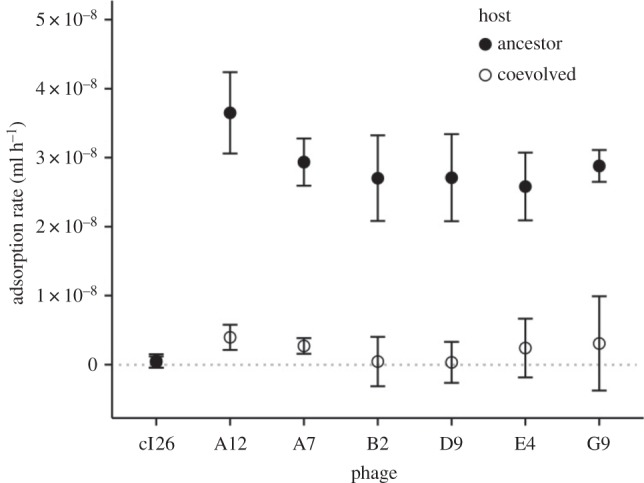
Virus and host coevolution alters adsorption rates. The evolved phages (A12, A7, B2, D9, E4 and G9), as a group, have increased adsorption rates relative to their ancestor (cI26) on both host types (see the text for statistical analyses). The coevolved host lowers the adsorption rates of the evolved phages relative to the ancestral host. No difference between the two hosts was detected for the ancestral phage; however, a difference was evident based on the growth rates of that phage. *n* = 4 for each evolved phage–host combination and *n* = 24 for each ancestor phage–host combination. Error bars are 95% confidence intervals (CIs).

To understand how the mutations in the phage's *J* gene and in the host's *malT* gene affected their interaction, we quantified the rate at which the ancestor and evolved intermediate phage genotypes adsorbed to both the ancestor and coevolved hosts (figure 1). The evolved intermediate phages as a group had higher adsorption rates than the ancestor phage on both the ancestral host (paired *t*-test, *t*_s_ = 18.893, d.f. = 5, *p* < 0.001) and coevolved host (paired *t*-test, *t*_s_ = 1.837, d.f. = 5, *p* = 0.063), although the latter difference was only marginally significant. Also as expected, the evolved intermediate phages had significantly lower adsorption rates on the coevolved host than on the ancestral host (paired *t*-test, *t*_s_ = 21.895, d.f. = 5, *p* < 0.001). These results indicate that the parasite and host populations engaged in a coevolutionary tug-of-war over the rate at which the parasites adsorb to and infect host cells. Mutations that increase the adsorption rate benefit the phage, and mutations that decrease the adsorption rate benefit the host. (We note that for some phage λ strains, the adsorption rate is influenced by a second gene, *stf*, that affects so-called side-tail fibres [26]. However, the phage strain that we used, cI26, is *stf*^–^ and lacks side-tail fibres. The cI26 genotype was generated by a 5996 bp deletion that includes the last 235 bp of the *stf* gene [10], so reversion to the *stf*^+^ allele is not possible.)

These results indicate that the adsorption rate was under strong selection during the experimental evolution of the phage λ populations. However, we were unable to resolve a difference in adsorption rates for the ancestral phage between the two hosts, because those rates were so low that they were at or even below our limit of detection. (In fact, the observed difference was opposite in direction to our hypothesis, and so the result of the *t*-test is *p* > 0.5.) This low resolution is similar to previous attempts to quantify the adsorption rate of *stf*^–^ phage λ to sensitive *E. coli* cells in a similar glucose environment, whereas phage λ adsorbs quite rapidly to cells grown on maltose, which induces LamB expression [18,26]. Under the glucose conditions used in our assays, the expression of LamB protein is under catabolite repression; these phage receptors are therefore scarce, and the absence of phage side-tail fibres also reduces the phage's adsorption rate. We observe that on the coevolved host, where LamB expression is further reduced by the host's *malT* mutation, adsorption is even more difficult to detect. Despite this limitation, our results demonstrate that all six evolved phage λ substantially improved their rate of adsorption to the scarce LamB molecules found on both the ancestral and coevolved hosts in the glucose environment (figure 1).

### (c) Mutations increase population growth rates of the intermediate phage genotypes

Selection has clearly favoured increased adsorption rate in the phage populations, but these improvements could, in principle, have harmful pleiotropic effects on other phage traits. Therefore, to quantify the full effect of the *J* alleles on phage fitness, we calculated the population growth rates of the ancestral and same six evolved phages during direct competitions for both the ancestral and coevolved hosts. If competition for LamB receptors drove the evolution of the *J* gene, then we also expect the evolved phages, with their faster adsorption rates, to initiate infections more quickly and have increased population growth rates and higher relative fitness. By performing the competitions on both cell types, we can also see how the host's coevolution affected selection on the phage.

**Figure 2.**
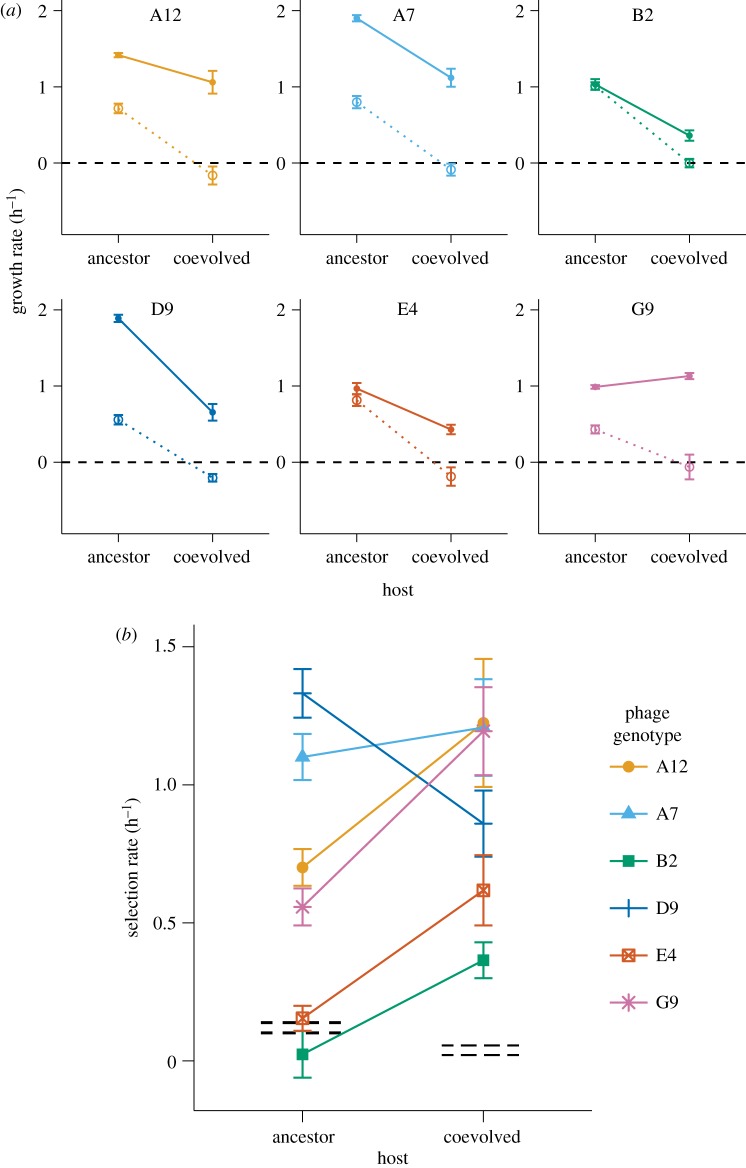
Effects of coevolution on the growth rates and fitness of phage λ. (*a*) Realized growth rates of ancestral (dashed lines) and evolved phage (solid lines) during direct competition for the ancestral and coevolved *E. coli* hosts. Phage genotypes A12, A7, B2, D9 and E4 grew faster on the ancestral than the evolved host (one-tailed *t*-tests, *p* < 0.05 after Bonferroni correction); G9 appeared to grow faster on the coevolved host, contrary to our expectation. (*b*) Selection rates, which provide a measure of the fitness of an evolved phage relative to the ancestral phage, on the ancestral and coevolved hosts. Selection rates are calculated as the difference in the growth rates of the evolved and ancestral phage using the data shown in panel (*a*). Four of the six evolved phage (A12, A7, D9 and G9) were significantly more fit than their ancestor on the ancestral host; all six were significantly more fit than their ancestor on the coevolved host. Also, four phage (A12, B2, E4 and G9) had significantly higher selection rates on the coevolved host than on the ancestral host (electronic supplementary material, table S2). The genetic marker used to distinguish phage competitors imposed a small fitness cost on the phage, and that cost differed between the two hosts; the black dashed lines show the 95% CIs for the selection rate when the unmarked and marked ancestral phages competed. (*a*) Growth rates and (*b*) selection rates. (Online version in colour.)

All six evolved intermediate phage genotypes grew faster than their ancestor (figure 2*a*, solid versus dotted lines) during competitions for the ancestral host, the coevolved host or both. Five of the six evolved phages (all except G9) grew more slowly on the coevolved *malT̄* host than on the ancestral host. The ancestral phage population grew measurably on the ancestral host (despite the low adsorption rate discussed above), even in the presence of the evolved phage genotypes, but it did not grow to any measurable extent on the coevolved host during the competitions.

### (d) Mutations improve relative fitness of the intermediate phage genotypes

The competitions between the evolved and ancestral phage genotypes allow us not only to calculate growth rates but also to quantify their relative fitness. As noted in the Material and methods section, we express relative fitness using selection rates calculated as the *difference* in realized growth rates of the evolved and ancestral phage, rather than as the *ratio* of their growth rates. We do so because the ancestral phage grows so poorly, if at all, on the coevolved host (figure 2*a*) that the ratio becomes noisy and even pathological (e.g. producing a negative value). The difference in growth rates, by contrast, is no more sensitive to errors in measuring the ancestor phage's growth rate than it is to errors in measuring the evolved genotype's growth rate.

By considering the growth rates of the evolved intermediate and ancestral phages together, we can assess whether the host evolution at the *malT* locus increased or reduced the strength of selection for the phage intermediates. Such data, in turn, offer insights into whether the host's coevolution promoted or impeded the evolution of the phage's ability to use the alternative OmpF receptor. Four of the six evolved intermediate phage genotypes (A12, A7, D9 and G9) were significantly more fit than their common ancestor on the ancestral host cells (figure 2*b*, ancestor host). All six evolved phage types were more fit than their ancestor when competing for the coevolved host cells (figure 2*b*, coevolved host). These data demonstrate that all six of the independently evolved phage genotypes had selective advantages, even *before* their descendants had gained the ability to use the OmpF receptor. Such benefits were previously hypothesized because, without them, it was difficult to understand how the first few mutations required for phage λ to evolve the ability to exploit the OmpF receptor had accumulated so quickly and repeatedly across many replicate populations [10]. Our results show that at least some of the intermediate steps that led to this new function increased the phage's adsorption rate and relative fitness. These mutations were thus favoured by selection, which helps to explain how the phage quickly and repeatedly achieved their innovation.

### (e) Host coevolution alters the adaptive landscape for the phage

In addition to showing that the intermediate phage genotypes were favoured by selection, the competition assays also reveal a parasite genotype by host genotype interaction. That is, the fitness of most of the tested phage genotypes (all except A7) relative to their ancestor depends significantly on the host genotype (figure 2*b*).

In four cases (A12, B2, E4 and G9), the selective advantages of the evolved phages were significantly greater during competition for the coevolved host cells (figure 2*b*). Selection favouring the evolved phage types was stronger on the coevolved host genotype despite the fact that phage growth was significantly slower on that host in three of these four cases (all except G9; figure 2*a*). Thus, in these four cases, host coevolution accelerated the evolution of phage lineages that were on the path leading to the new ability to use the OmpF receptor (figure 2*b*). By contrast, evolved phage D9 had a significantly greater advantage on the ancestral host, and phage A7 was approximately equally fit on the ancestral and coevolved hosts (figure 2*b*).

Taken together, our results reveal important similarities and substantial variation among the six intermediate phage types examined here. Most importantly, and consistent with the general hypothesis that the intermediates reached high frequencies because the mutations they carried conferred selective advantages, all six phage were significantly more fit than their ancestor when competing for the ancestral host, the coevolved host or both. The results also tend to support, though not in every case, the specific hypothesis that *host* coevolution pushed the evolving phage populations along paths that accelerated the rise of the intermediate types and hence the subsequent emergence of phage with the novel ability to exploit the OmpF receptor. This latter trend is consistent with theory that predicts coevolution can drive innovation by altering the shape of fitness landscapes in ways that favour new phenotypes [27–29].

Several other studies also support the hypothesis that coevolution can promote innovation. Populations of phage *ϕ*2 explored genomic space more broadly when they adapted to coevolving hosts than when they adapted to a static host [30]. When phage M1 was sequentially presented with hosts expressing potential receptors that were increasingly different from its usual receptor, the phage evolved the ability to use a protein not previously known to have that capacity [31]. Also, experiments with digital hosts and parasites, analogous in some respects to bacteria and phage, showed that coevolution generated negative frequency-dependent selection, which deformed fitness landscapes in ways that favoured the evolution of new, more complex traits [32]. Together with these studies, our results show that the adaptive landscape of a virus or other parasite can be reshaped by the coevolution of its hosts.

### (f) Relationships between adsorption, growth and selection rates

In general, the increased adsorption rates of the evolved phages (figure 1) resulted in faster population growth (figure 2*a*) and higher fitness (figure 2*b*) relative to the ancestral phage on one or both host genotypes. However, there are some apparent discrepancies between the various performance measures. The most striking cases are B2 and E4; both have much higher adsorption rates than their ancestor on the ancestral host, but they have little or no growth or fitness advantage on that host. There are several potential explanations for these differences. First, there could be trade-offs between adsorption rate and other phage life-history traits [19,33]. For example, some mutations might cause phage particles to be less stable and decay more rapidly. We measured decay rates in cell-free media in assays performed alongside the adsorption-rate assays, subtracting the decay rate from the overall rate of phage loss in the presence of host cells, and then dividing by the host-cell density, to obtain the adsorption rates reported in figure 1. The decay rates were generally small (more than an order of magnitude lower than the overall rate of phage loss by adsorption to the ancestral host for the six evolved phage), and there were no large differences among the phage genotypes in their decay rates (electronic supplementary material, figure S1). However, decay rates might also depend on the density of receptors in the cell debris that is generated as phage replicate and lyse their hosts, which would not be reflected in decay rates measured in cell-free media. Second, trophic interactions, including those between phage and bacteria, can generate feedbacks that may complicate interpretation of the dynamics. For example, a faster-growing phage population kills more host cells, which would otherwise replicate and provide more prey, than does a slower-growing phage population. Third, the competition protocol might produce some complications. In particular, using the *lacZ* marker system for the phage required removal of the host cells (which are *lacZ*^+^) from the samples prior to counting the phage competitors. (If the cells were not removed, they would form a blue lawn on the plates, preventing differentiation of the evolved and marked ancestral phage.) The removal of host cells also removes phages that have initiated but not yet completed infections, which might lead to underestimating the growth rate and fitness of phage with high adsorption rates. Despite these and perhaps other complications, however, the adsorption, growth and selection rates collectively show that the evolving phage populations were under intense selection to improve their adsorption to the LamB receptor both before and after their hosts evolved reduced expression of that protein.

### (g) Implications of ecological interactions for predicting evolution

It is important to account for the effects of ecological interactions on the relative fitness of different genotypes if one is to understand and even predict evolutionary changes [34,35]. In this study, we examined how the tug-of-war over a virus's adsorption rate mediated the coevolutionary arms race between a phage and its bacterial host. Additionally, we demonstrated how the host's coevolution led to varied, and sometimes even opposite, effects on the relative fitness of different virus genotypes (figure 2*b*). Predicting viral evolution may therefore depend on knowing the genetic states of both the host and parasite populations and understanding how those states affect fitness. Scaling this understanding to more diverse and complex ecosystems is challenging [36], but it may well be necessary to predict such phenomena as disease emergence. It is not difficult to imagine that coevolution might sometimes *block* certain adaptive paths. However, our results show that coevolution can produce dynamic fitness landscapes in which populations are *more* evolvable than they would otherwise be. As a consequence, coevolutionary dynamics may promote some key innovations and other hard-to-reach adaptations.

## Supplementary Material

Burmeister et al. Supplementary Material for Host Coevolution Alters the Adaptive Landscape of a Virus

## Data accessibility

Additional methods, two tables and one figure are included as the electronic supplementary material. Data and analysis scripts were deposited in Dryad: http://datadryad.org/resource/doi:10.5061/dryad.2tj43 [37].
