# Supplementary material for: Host coevolution alters the adaptive landscape of a virus
Source: Proc Biol Sci. 2016 Sep 28;283(1839):20161528. doi: 10.1098/rspb.2016.1528 (PMC5046904; doi:10.1098/rspb.2016.1528)

Host coevolution alters the adaptive landscape of a virus

Alita R. Burmeister, Richard E. Lenski, and Justin R. Meyer

Proc. R. Soc. B 20161528. http://dx.doi.org/10.1098/rspb.2016.1528

Received: 7 July 2016
Accepted: 2 September 2016

SUPPLEMENTARY MATERIALS

Supplementary Materials and Methods

(a) Duration of fitness assays

The derived phage λ genotypes used in this study evolved in serial batch culture with daily transfers [10]. In principle, we would have preferred to measure fitness over the same 24-h period in the competitive fitness assays. However, preliminary experiments indicated that the fitness advantages of the evolved genotypes were often so great that they overwhelmed the ancestral phage over the 24-h transfer cycle, making it difficult to detect the ancestral type in the final plate counts. We tried plating higher concentrations of phage, and that allowed us to detect some ancestral plaques, but these plates were then too crowded to count reliably. We also tried (i) increasing the initial frequency of the ancestor and (ii) reducing the duration of the competitions to 4 h, before settling on the 8-h assays as the most reliable.

A second obstacle that arose during 24-h phage competitions was that Mal^- phage-resistant mutant hosts often evolved from the Mal^+ ancestral strain, REL606, and reached high frequencies over that period. This rapid host evolution would therefore confound the comparisons of phage fitness values measured on the ancestral and coevolved hosts. We monitored the frequency of Mal^- bacteria in all phage competitions on the ancestral host to ensure that these mutants did not interfere with our estimates. To do so, we plated cells at the end of each replicate competition onto tetrazolium maltose indicator agar; Mal^- and Mal^+ cells produce red and white colonies, respectively, on that medium [10]. We found that the frequency of Mal^- cells remained <5% in most 8-h competitions, whereas their frequency rose to >50% in most preliminary competitions that ran for 24 h.

Table S1. Bacterial strains, phage strains, and PCR primer sequences used in this study.

Bacterial strains

Strain	Source	Relevant Characteristics
**REL606**	Ancestor host	λ susceptible, *lamB*$^{+}$, *ompF*$^{+}$, *lac*$^{+}$
**EcC4**	Evolved from REL606	Partial λ resistance, *malT*$^{-}$, *lamB*$^{+}$, *ompF*$^{+}$, *lac*$^{+}$
**DH5α**	Unrelated plating host	Used for plating marked ancestor λ, *lacZα*$^{-}$

Phage strains

Strain	Source	Relevant Characteristics
**cI26**	Ancestor phage λ	Strictly lytic (frameshift mutation in *cI*), Δ*stf*, no mutations in *J* gene
**λ_{lacZ}**	Marked ancestor phage λ	Derivative of cI26 carrying *lacZα* gene
**A12**	Population A12, day 10	6 *J* mutations, 1 of 4 required for infection via OmpF
**A7**	Population A7, day 10	4 *J* mutations, 2 of 4 required for infection via OmpF
**B2**	Population B2, day 13	3 *J* mutations, 2 of 4 required for infection via OmpF
**D9**	Population D9, day 8	4 *J* mutations, 2 of 4 required for infection via OmpF
**E4**	Population E4, day 13	3 *J* mutations, 2 of 4 required for infection via OmpF
**G9**	Population G9, day 11	5 *J* mutations, 2 of 4 required for infection via OmpF

PCR primer sequences

Primer	Location	Sequence
**RL0932**	Upstream of λ *J* gene	Forward, 5’ CTGCGGGCGGTTTTGTCATT 3’
**RL0933**	Downstream of λ *J* gene	Reverse, 5’ ACGTATCCTCCCCGGTCATCACT 3’
**RL1031**	Internal to λ *J* gene	Reverse, 5’ GACGCCGGACAGCACCACAGACC 3’
**RL1032**	Internal to λ *J* gene	Reverse, 5’ CGCCCTGAAGGACCGCCATAAT 3’
**RL1033**	Internal to λ *J* gene	Reverse, 5’ CAGCATGTGCCGGAAAAAGAGG 3’
**RL1034**	Internal to λ *J* gene	Reverse, 5’ CTTTTATATCCGCAGTGTGAAC 3’
**RL1035**	Internal to λ *J* gene	Reverse, 5’ ATAGCTGAAAACTGTACGATAAAC 3’
**cI rev**	In *cro* upstream of cI	Reverse, 5’GCGGGGTTATTTATGCTGTTGTT 3’
**cI for**	In *rexA* downstream of cI	Forward, 5’AGCGCTTATCTTTCCCTTTATTTT 3’

Table S2. Calculated *p*-values for two methods used to determine whether the fitness of the evolved phage genotypes depended on the host genotype. Values in the first column were not corrected for the fitness effects of the *lacZ* marker, which differed between the two host types, whereas those in the second column were adjusted by subtracting the average marker effect on each host before determining significance. Adjusting for the marker effect changed the *p*-values slightly, but it did not affect the overall interpretation of the results. The asterisk indicates that the observed difference went in the opposite direction to that hypothesized.

Genotype	Uncorrected	Corrected
**A12**	< 0.001	< 0.001
**A7**	0.107	0.019
**B2**	< 0.001	< 0.001
**D9**	> 0.5*	> 0.5*
**E4**	< 0.001	< 0.001
**G9**	< 0.001	< 0.001

Figure S1. Decay rates of six evolved phage genotypes and their ancestor, cI26, in media without any host cells. These decay rates were subtracted from the rates of phage loss in the presence of host cells to obtain the adsorption rates shown in Figure 1.

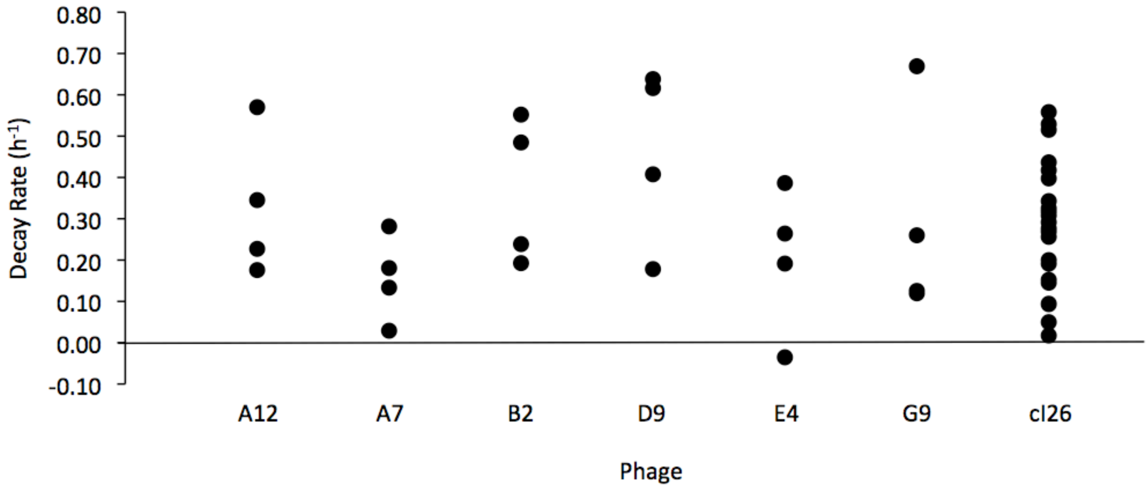

Supplement: Burmeister et al. Supplementary Material for Host Coevolution Alters the Adaptive Landscape of a Virus [file rspb20161528supp1.pdf]
